# A direct contact pig influenza challenge model for assessing protective efficacy of monoclonal antibodies

**DOI:** 10.3389/fimmu.2023.1229051

**Published:** 2023-10-27

**Authors:** Adam McNee, Daryll Vanover, Pramila Rijal, Basudev Paudyal, Fabian Z. X. Lean, Ronan MacLoughlin, Alejandro Núñez, Alain Townsend, Philip J. Santangelo, Elma Tchilian

**Affiliations:** ^1^ Host Responses, The Pirbright Institute, Pirbright, United Kingdom; ^2^ Wallace H. Coulter Department of Biomedical Engineering, Emory University, Atlanta, GA, United States; ^3^ Weatherall Institute of Molecular Medicine, University of Oxford, Oxford, United Kingdom; ^4^ Department of Pathology, Animal and Plant Health Agency (APHA)-Weybridge, Addlestone, United Kingdom; ^5^ Research and Development, Science and Emerging Technologies, Aerogen Ltd, Galway, Ireland

**Keywords:** influenza, pig, aerosol delivery, contact challenge, transmission, nasal shedding, 2-12C monoclonal antibody

## Abstract

Monoclonal antibodies (mAbs) can be used to complement immunization for the therapy of influenza virus infection. We have established the pig, a natural large animal host for influenza A, with many physiological, immunological, and anatomical similarities to humans, as an appropriate model for testing mAbs. We have evaluated the protective efficacy of the strongly neutralizing human anti-hemagglutinin mAb, 2-12C in the pig influenza model. Intravenous administration of recombinant 2-12C reduced virus load and lung pathology, however, it did not prevent virus nasal shedding and, consequently, transmission. This may be because the pigs were directly infected intranasally with a high dose of the H1N1pdm09 virus. To address this, we developed a contact challenge model in which the animals were given 2-12C and one day later co-housed with donor pigs previously infected intra-nasally with H1N1pdm09. 2-12C pre-treatment completely prevented infection. We also administered a lower dose of 2-12C by aerosol to the respiratory tract, but this did not prevent shedding in the direct challenge model, although it abolished lung infection. We propose that the direct contact challenge model of pig influenza may be useful for evaluating candidate mAbs and emerging delivery platforms prior to clinical trials.

## 1 Introduction

Influenza virus infection is a major health threat to humans and livestock, causing substantial mortality and morbidity. Monoclonal antibodies (mAbs) can provide immediate immunity and augment existing vaccines against seasonal and pandemic influenza infection. Prophylactic and therapeutic administration of neutralizing mAbs against conserved epitopes of the hemagglutinin (HA) stem and head and mAbs against the neuraminidase have been shown to be effective in mice and ferret models (1–6). We have established a robust and reproducible pig influenza challenge model to evaluate mAb delivery platforms (7).

The pig is a large animal natural host for similar influenza subtypes as human seasonal strains, with the further advantage that the H1N1pdm09 (pH1N1) virus circulates in both pigs and humans (8). Pigs can also be a source of novel viruses with pandemic potential (9). Pigs have a longer life span and are genetically, immunologically, physiologically, and anatomically more like humans than small laboratory animals (10–12). Pigs exhibit similar clinical manifestations and pathogenesis when infected with influenza viruses, making them an excellent model to study immunity to influenza. Furthermore, the distribution of sialic acid receptors in the respiratory tract of pigs and humans is similar. We have shown that porcine immune responses following infection with pH1N1 influenza virus are similar to those induced in humans (13).

We identified a protective human anti-hemagglutinin (HA) specific mAb, 2-12C, which can be used as a positive control to benchmark other mAb candidates and delivery platforms (7, 14). When recombinant 2-12C mAb was given at 15 mg/kg intravenously 24 hours before a pH1N1 challenge, it significantly reduced viral load and lung pathology. However, although it significantly reduced viral load, it did not prevent nasal viral shedding and, consequently, transmission. Similar results were observed with porcine anti-influenza mAbs that we isolated from pH1N1 experimentally infected pigs. Prophylactic delivery of the strongly neutralizing porcine mAbs – pb 27 or pb18 at 15mg/kg and 10 mg/kg, respectively, significantly reduced viral load in nasal swabs, bronchoalveolar lavage (BAL), and lung, but did not completely eliminate viral shedding (13, 15). One reason that the pigs continued to shed the virus may be because they were directly inoculated intranasally with a high dose of the pH1N1 virus.

To address this, we further developed the pig model to evaluate whether 2-12C prophylactic treatment prevents nasal viral shedding when the treated pigs are put in contact with previously infected pigs, a situation that more closely resembles natural infection. Since the influenza virus targets epithelial cells of the upper and lower respiratory tracts, we also wished to determine if mucosal delivery of 2-12C to the respiratory tract may be more effective in preventing nasal shedding than intravenous administration.

## 2 Materials and methods

### 2.1 mAb preparation

The anti-influenza HA1 human IgG1 mAb 2–12C was produced by Absolute Ab Ltd (Redcar, U.K.). It was dissolved in 25 mM histidine, 150 mM NaCl, and 0.02% Tween P80 (pH 6) diluent.

### 2.2 Influenza infection studies in pigs

All experiments were approved by the ethical review processes at the Pirbright Institute and Animal and Plant Health Agency (APHA) and conducted according to the U.K. Government Animal (Scientific Procedures) Act 1986 supported by Project Licenses P47CE0FF2 and PP2064443. Three influenza challenge studies were carried out.

For the first direct influenza challenge experiment, 10 5-week-old Landrace x Hampshire cross female pigs were obtained from a commercial high-health status herd. The pigs weighed between 9 and 13 kg (average 11.8 kg). The animals were screened for the absence of influenza A virus antibody by hemagglutination inhibition using four swine influenza virus antigens from H1N1pdm09, H1N2, H3N2, and avian-like H1N1. Pigs were randomized into two groups of five pigs: the first group was given 15 mg/kg 2-12C mAb intravenously (I.V.) and the second group was an untreated control. The rec 2-12C was administered to the ear vein of animals sedated with 4.4mg/kg (Zoletil, Virbac, UK) and 0.04 mg/kg medetomidine (Domitor, Orion Pharma, Finland). After 24 hours of the 2-12C administration, all pigs were inoculated intranasally with 3 x 10^6^ PFU of A/swine/England/1353/2009 (pH1N1) MDCK grown virus in a total of 2 ml (1 ml per nostril) using a mucosal atomization device (MAD, Wolfe-Tory Medical) (**Table 1**). After the pH1N1 challenge, daily nasal swabs were collected for 4 days to assess the virus load by plaque assays. Blood samples were collected at days 0, 1, 3, and 4 post-infection. The pigs were humanely killed 4 days post pH1N1 challenge, and blood, bronchoalveolar lavage (BAL), and lung samples were collected to measure virus load and 2-12C titers.

**Table 1:** Overview of influenza challenge studies.

Experiment	Method of challenge	Treatment groups
1. Direct influenza challenge	Direct intranasal inoculation using a mucosal atomization device	1. Control untreated 2. 2-12C intravenous
2. Contact influenza challenge	Co-housing with influenza-infected donor pigs	1. Control untreated 2. 2-12C intravenous
3. Direct influenza challenge	Direct intranasal inoculation using a mucosal atomization device	1. Control untreated 2. 2-12C intravenous 3. 2-12C aerosol

For the contact influenza challenge experiment, 20 5-week-old Landrace x Hampshire cross female influenza-free pigs were sourced as described above. The pigs weighed between 8 and 13 kg (average 11.3 kg). Pigs were randomized into the following three groups: 10 donor pigs were inoculated with 6 x 10^6^ PFU of pH1N1 intranasally using MAD (1 ml per nostril); 5 recipient pigs were given 15 mg/kg 2-12C I.V. following sedation as above, and 5 pigs were untreated recipient controls. After 24 hours of the 2-12C administration, the five recipient 2-12C pigs were put in contact with five donor pigs infected 48 h previously with pH1N1. Similarly, the five control untreated recipient pigs were put in contact in a separate room with five previously infected donor pigs. The pigs were co-housed for 5 days, after which the donor pigs were removed and culled. The recipient pigs were culled after a further 2 days or 7 days after the contact (**Table 1**). Daily nasal swabs and blood samples were collected from the recipient pigs for 7 days post pH1N1 infection.

The third direct influenza challenge study, which evaluated aerosol delivery of 2-12C, used 15 5-week-old Landrace x Hampshire cross female influenza-free pigs that were acquired as described above. The pigs weighed between 8 and 14 kg (average 11.8 kg). Pigs were randomized into three groups of five pigs as follows: 1) 2-12C administered I.V. at 15 mg/kg following sedation as above; 2) 2-12C administered by aerosol, and 3) untreated controls (**Table 1**). Aerosol delivery was performed using an Aerogen Solo vibrating mesh nebulizer with a ProX controller (Aerogen, Dangan, Galway, Ireland) attached to a bespoke face mask held over the animal’s nose and mouth (16, 17). Using laser diffraction and cascade impaction, the droplet size for the nebulizer was recorded as 4.5 microns volumetric median diameter (17, 18). For aerosol delivery, 2 ml of 15 mg/ml 2-12C was administered to sedated pigs over 5-10 minutes. After 24 hours of the 2-12C administration, all animals were inoculated with PFUs of pH1N1 in 2 ml (1 ml per nostril) using a MAD. Daily nasal swabs were collected for 4 days, and blood samples were collected at days 0, 1, 3, and 4 post pH1N1 infection. The pigs were humanely culled 4 days post-pH1N1 challenge.

Clinical signs (temperature, state of breathing, coughing, nasal discharge, appetite, and altered behavior) observed in the three experiments were mild and none of the pigs developed moderate or severe disease.

### 2.3 Pathological and histopathological examination of lungs

Gross and histopathological analyses were performed as previously described (7). Both the dorsum and ventrum of the lungs were photographed following extraction from the thorax. Macroscopic pathology was blindly scored by a veterinary pathologist as previously reported (19). Left cranial, middle, and caudal lung lobes were fixed in 10% neutral-buffered formalin and processed by a routine histological method. Formalin-fixed paraffin wax–embedded tissues were sectioned into 4-mm thickness and stained with H&E and immunohistochemistry (IHC) against influenza A virus nucleoprotein (NP) (20). Lung histopathology and viral IHC were assessed by a veterinary pathologist blinded to the treatment group. The pulmonary histopathology was scored using five parameters: necrosis of the bronchiolar epithelium, airway inflammation, perivascular/bronchiolar cuffing, alveolar exudates, and septal inflammation. Each parameter was scored on a scale of 0–4 for each lung lobe. The scores were then summed to give a score ranging from 0–20 per lung lobe and a total animal score (from 3 lung lobes) from 0–60 (21). The pulmonary IHC was scored separately for the bronchioles and alveoli, on a scale of 0-4 for each lung lobe (0: no immunolabelling, 1: less than 5% of cells immunolabelled, 2: greater than 5% and less than 25% of cells immunolabelled, 3: greater than 25% and less than 50% of cells immunolabelled, and 4: greater than 50% of cells immunolabelled). The scores were then summed to give a score ranging from 0–8 per lung lobe and a total animal score (from 3 lung lobes) from 0–24.

### 2.4 Tissue sample processing

Two nasal swabs (both in two nostrils) were taken at the indicated time points and placed into 2 ml of virus transport medium 199 (Sigma-Aldrich, St. Louis, MO) supplemented with 25 mM HEPES, 0.035% sodium bicarbonate, 0.5% BSA, 100 IU/ml penicillin, 100 mg/ml streptomycin, and 0.25 mg/ml nystatin. The samples were vortexed, centrifuged to remove debris, and stored at -80˚C for subsequent virus titration. Blood samples were collected at the start of the study (prior to 2-12C administration) and at the indicated times post-mAb delivery and influenza virus challenge. Blood was allowed to clot before centrifugation at 900 x g for 5 minutes, the serum was removed, aliquoted, and frozen for analysis of antibody titers. BAL was collected from the entire left lung with 100 ml of PBS into the lung, and 50 ml of fluid was recovered. BAL samples were centrifuged at 500 x g for 5 minutes and the supernatant was removed, aliquoted, and frozen for further analysis of viral load and antibody titers. Accessory lung lobes were taken from all pigs at the postmortem stage, and the tissues were homogenized using a Miltenyi cell dissociator at a ratio of 1g of material to 1 ml of RPMI media. The cell suspension was centrifuged at 500 x g for 5 minutes before the supernatant was removed, aliquoted, and frozen for further analysis of viral load by plaque assays on Madin–Darby canine kidney (MDCK) cells and antibody titers (7). The limit of detection for the plaque assay was 10 plaque-forming units (pfu)/ml.

### 2.5 Microneutralization and ELISA assays

Neutralizing Ab titers against pH1N1 were determined in serum and BAL fluid as previously described (7). Antibody titers in serum and BAL fluid were determined by ELISA against recombinant HA protein of A/Eng/195/2009 hemagglutinin as previously described (14).

### 2.6 Statistical analysis

Statistical analyses were performed using GraphPad Prism 9.2.0 (GraphPad Software, San Diego, CA, United States). The data sets were first analyzed for normality and then subjected to either a t-test or one-way ANOVA with Sidak’s multiple comparisons test when normally distributed or to a Mann-Whitney test or Kruskal-Wallis test and Dunn’s multiple comparisons test when normality was not achieved (the figure legends state the data sets/graphs that were not normally distributed). Significant differences were either presented on each graph or listed in **Table 1** (*p < 0.05, **p < 0.01, ***p < 0.001, ****p < 0.0001).

## 3 Results

### 3.1 2-12C does not prevent nasal shedding following a direct influenza challenge

**Figure 1:**
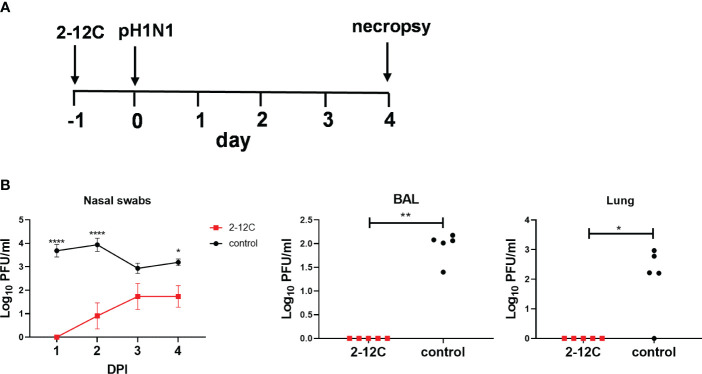
Experimental design and viral load. In the treatment group, recombinant 2-12C was administered intravenously to pigs, whereas the control group remained untreated. Both groups were infected with the pH1N1 virus 24 hours later. Nasal swabs (NS) were taken at 1-, 2-, 3-, and 4-days post-infection (DPI), and pigs were sacrificed at 4 DPI **(A)**. Viral titers in nasal swabs, accessory lung lobe (Lung), and BAL were determined by plaque assay **(B)**. Viral shedding in NS is represented as the mean of the five pigs on each day and the significance versus untreated control is indicated by asterisks. Each data point in BAL and lung represents an individual pig, and the bars show the mean. Viral titers were analyzed using multiple unpaired Mann-Whitney tests (BAL and Lung), the two-way ANOVA test (NS), and Dunn’s multiple comparisons test. Asterisks denote significant differences. *, *p*<0.05; **, *p*<0.01; ****, *p*<0.0001 versus indicated control groups.

We have previously shown that prophylactic administration of the strongly neutralizing human mAb, 2-12C at 15 mg/kg significantly reduced viral load in BAL and lung and lung pathology but did not prevent nasal shedding (7). To ensure that 2-12C was still capable of reducing viral load in the direct infectious model we carried the experiment shown in **Figure 1A** as a positive control for the subsequent contact challenge experiment. We administered 15 mg/kg of 2-12C intravenously (I.V.) and the control groups were left untreated. After 24 hours, the pigs were challenged with pandemic swine H1N1 isolate and A/swine/England/1353/2009 (pH1N1), and 4 days later, they were culled to assess viral load and pathology (**Figure 1A**). 2-12C significantly reduced the viral load in nasal swabs over the 4 days in animals treated with 2-12C as determined by the area under the curve (AUC) compared to controls (*p* = 0.016), but shedding was not eliminated (**Figure 1B**). No virus was detected in the BAL and lungs in the 2-12C group at 4 days post-infection (DPI).

**Figure 2:**
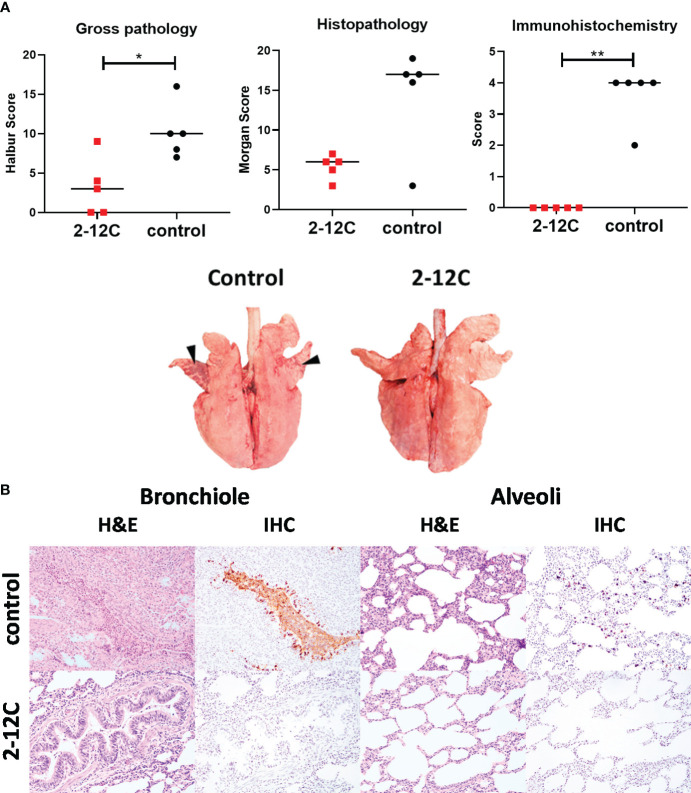
Lung pathology. Recombinant 2-12C was administered intravenously to pigs, which were infected with pH1N1 virus 24 h later along with untreated controls. The animals were culled at 4 DPI, and lungs were scored for the appearance of gross and histopathological lesions. The score for each individual in a group and the group means are shown **(A)**. Representative gross pathology, histopathology (H&E staining; original magnification 3100), and immunohistochemical NP staining (original magnification 3200) for each group are shown **(B)**. Pathology scores were analyzed by unpaired t-tests. Asterisks denote significant differences. *, *p*<0.05, **, *p*<0.01 versus indicated control groups.

Macroscopic and microscopic lung pathology were significantly reduced in the 2-12C treated pigs compared to the control group (**Figure 2A**). Histopathological analysis revealed that the 2-12C group had occasional intrabronchiolar exudation and neutrophilic exocytosis and that alveoli were generally unremarkable and virus antigens were not detected. In contrast, the control group exhibited a moderate to large number of neutrophilic exudates within the bronchiolar lumen, mild leucocytic infiltration within the alveolar walls, and a moderate amount of virus antigens were detected, which were frequently co-localized to areas of bronchiolar and alveolar lesions (**Figure 2B**).

**Figure 3:**
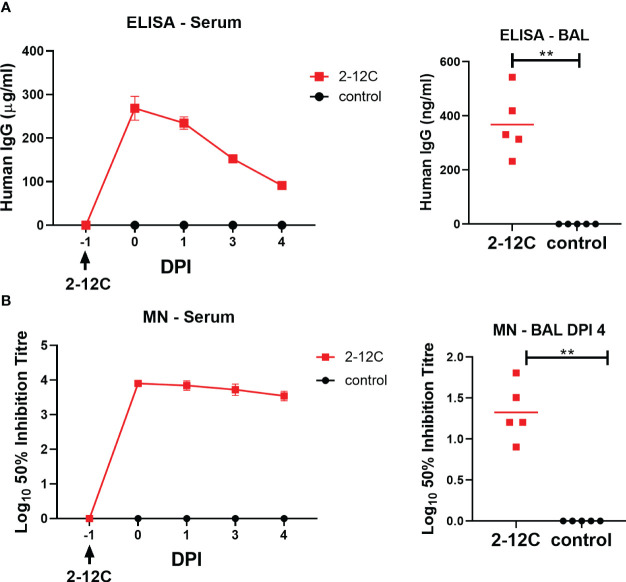
Concentration and neutralizing titers of 2–12C in serum and BAL. H1 HA-specific IgG in serum, BAL at 4 DPI, and nasal swabs (NS) at the indicated DPI **(A)**. 50% neutralization titers against pH1N1 in the serum and BAL at 4 DPI **(B)**. Symbols represent an individual pig within the indicated group, and lines represent the mean. Data were analyzed for serum as a Mann-Whitney test for BAL. Asterisks denote significant differences. **, *p*<0.01 versus indicated control groups.

The concentration of administered 2-12C in serum at 0, 1, 3, and 4 DPI was determined by ELISA using recombinant HA from A/Eng/195/2009. Peak concentrations of 268 µg/ml were detected at 24 hours after administration. A decline in serum mAb concentrations was observed over the next 4 days to 91.2 µg/ml (**Figure 3A**). BAL samples at 4 DPI showed the presence of 2-12C at 367.7 ng/ml at 4 DPI. Neutralizing activity decreased in serum and BAL showing average 50% inhibition titers of 1:3,600 and 1:27, respectively, at 4 DPI (**Figure 3B**).

The data collectively demonstrate that the prophylactic administration of recombinant 2-12C at 15 mg/kg significantly reduced lung viral load and pathology. In addition, although nasal shedding also decreased it was not prevented, in agreement with our previous studies.

### 3.2 2-12C prevents nasal shedding following contact influenza challenge

**Figure 4:**
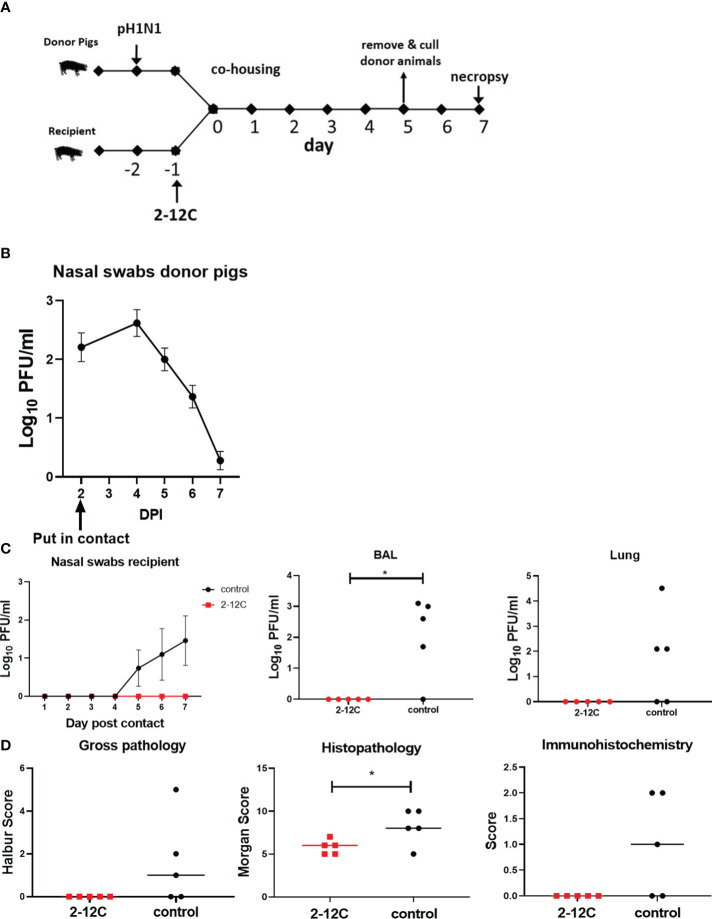
Experimental design, viral load, and pathology after contact challenge. Recipient pigs were given rec 2-12C and 24 hours later co-housed with donor pigs previously infected with pH1N1. Similarly, the five control untreated recipient pigs were put in contact with five previously infected donor pigs. The pigs were co-housed for 5 days and the recipient pigs were culled after a further 2 days or 7 days after the contact. Nasal swabs (NS) were sampled each day until sacrifice at 7 days post contact **(A)**. Viral shedding in NS from donor **(B)** and recipient pigs **(C)** was analyzed at the indicated time points post-infection or contact, respectively. Recipient pig viral shedding in BAL and Lung was determined at day 7 post contact. NS are represented as the mean of ten or five pigs for donor and recipient pigs, respectively. Lung gross pathology, histopathology and immunohistochemistry scores for each individual animal in a group and the group means are shown **(D)** Each data point in BAL and lung represents an individual pig, and the bars show the mean. Viral titers were analyzed using the Mann-Whitney test, *, *p*<0.05 versus indicated control groups.

We next wanted to determine if nasal shedding could be abolished after a contact influenza challenge that better simulates natural infection. The recipient animals were given either 2-12C I.V. at 15 mg/kg or left untreated. One day later the recipient animals were co-housed with donor pigs directly intranasally infected 2 days previously with pH1N1. The donor pigs were removed after 5 days and culled, and the recipient pigs were culled 7 days after contact to assess virus load and lung pathology (**Figure 4A**). Donor pigs shed virus in the first 7 days, confirming the successful infection of all animals and ensuring that recipient pigs were in contact with donors at the height of viral shedding (**Figure 4B**) No virus was detected in the nasal swabs of 2-12C treated recipient pigs (**Figure 4C**). In contrast, three out of the five untreated recipient controls shed virus by day 5 post contact. No virus was detected in the lung or BAL of the 2-12C recipients, while 4 out of the 5 control recipients had the virus detected in the BAL and 3 out of 5 had the virus detected in the lungs. As with the direct challenge, the control recipient group had a significantly higher histopathology score compared to 2-12C (**Figure 4D**) (**Supplementary Figure 1**). Histology revealed mild broncho interstitial pneumonia in the untreated controls with viral antigens present in bronchiole and alveoli. In contrast, no microscopic pathology nor virus antigens were detected in the 2-12C treated recipient animals.

**Figure 5:**
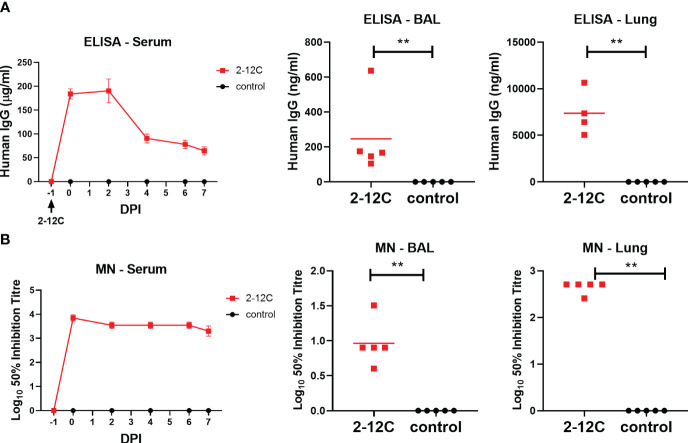
Concentration and neutralizing titers of 2-12C in serum and respiratory tissues. H1 HA-specific IgG in serum at the specified time points as well as BAL and Lung at 7 days post contact **(A)**. 50% neutralization titers against pH1N1 in the serum, BAL, and Lung **(B)**. Symbols represent an individual pig within the indicated group and lines represent the mean. Data were analyzed using the Mann-Whitney test. Asterisks denote significant differences **, *p*<0.01 versus indicated control groups.

The concentration of 2-12C in serum was determined daily after the contact challenge. 2-12C was detected at 184 µg/ml 24 hours after administration, which declined to 70.6 µg/ml by day 7 post contact. BAL and lung also showed the presence of 2-12C, at 0, 25 µg/ml and 7.4 µg/ml, respectively, at 7 days post contact (**Figure 5A**). Neutralizing activity was reduced over time in serum, BAL, and lung with respective 50% inhibition titers of 1:2,200, 1:12, and 1:460, respectively, at 7 days post contact (**Figure 5B**).

Overall, the data indicates that prophylactic administration of 2-12C in a direct contact challenge, which best mimics natural exposure, can prevent infection, eliminate shedding, and, by implication, interrupt onward transmission.

### 3.3 Aerosol delivery of 2-12C does not prevent nasal shedding following a direct influenza challenge

**Figure 6:**
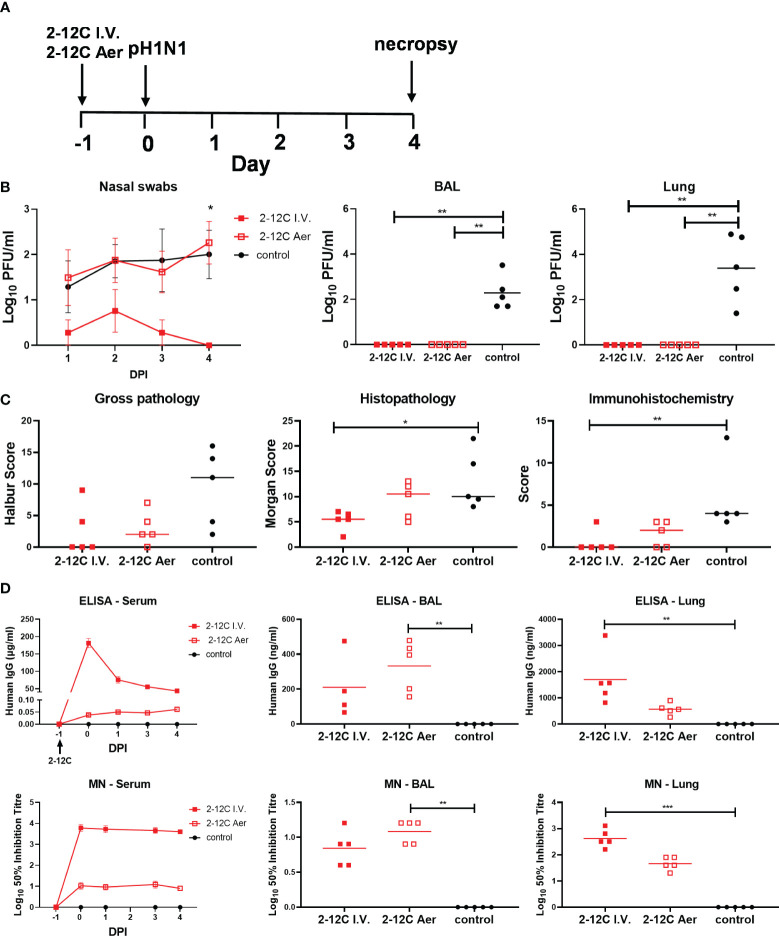
Experimental design and viral load. Recombinant 2-12C was administered intravenously or by aerosol to pigs 24 hours prior to inoculation with pH1N1 virus. Nasal swabs (NS) were taken at 1, 2, 3, and 4 DPI. **(A)**. Viral titers in nasal swabs, accessory lung lobe (Lung), and BAL were determined by plaque assay **(B)**. The animals were culled at 4 DPI, and lungs were scored for the appearance of gross, histopathological lesions and NP staining by immunohistochemistry. The score for each individual in a group and the group means are shown **(C)**. Concentration and neutralizing titer of 2–12C in serum, BAL, and lung. H1 HA-specific IgG in serum, BAL at 4 DPI, and nasal swabs (NS) at the indicated DPI. 50% neutralization titers against pH1N1 in serum and BAL at 4 DPI **(D)**. Symbols represent an individual pig within the indicated group and lines represent the mean. Data were analyzed using the Mann-Whitney test **(C)** and Kruskal-Wallis test (B + C) with Dunn’s multiple comparisons tests. Asterisks denote significant differences *, *p*<0.05; **, *p*<0.01; ***, *p*<0.001 versus indicated control groups.

We next hypothesized that delivering 2-12C to the respiratory tract might inhibit viral replication and shedding at the site of infection. We administered 2 ml of 15 mg/ml 2-12C by aerosol (Aer) using a vibrating mesh nebulizer and bespoke mask (16, 17). Aerosol delivery was compared to 2-12C administered I.V. at 15 mg/kg and untreated controls. Using gamma scintigraphy with technetium complexed with diethylenetriaminepentaacetic acid, we had previously established that aerosol delivers approximately 20-30% of the dose (17). Therefore we can assume that at least 150 times less 2-12C was delivered by Aer compared to I.V. The pigs were challenged with pH1N1 24 hours after 2-12C administration and culled 4 days later (**Figure 6A**). As before, I.V. administration of 2-12C significantly reduced but did not prevent virus shedding in nasal swabs following challenge over 4 days (*p* = 0.0125). The Aer delivered 2-12C showed no impact on nasal viral load. However, both I.V. and Aer delivery abolished viral load in BAL and lung as determined by plaque assays (**Figure 6B**). Whilst both the I.V. and Aer groups showed a trend of a reduced gross lesion score, only the I.V. group achieved statistical reduction on histopathology and IHC score (**Figure 6C**; **Supplementary Figure 2**). I.V. administration resulted in a greater titer of 2-12C in the serum (181.2 µg/ml) and lung (1.7 µg/ml) 24 hours post treatments compared to Aer, which had 0.04 µg/ml and 0.57 µg/ml, respectively. Aer, in contrast, achieved higher 2-12C titer in the BAL, at 0.33 µg/ml, compared to the I.V. group, at 0.21 µg/ml (**Figure 6D**). Virus neutralization was comparable to the 2-12C ELISA titers, with greater concentrations of mAb corresponding with higher neutralizing titers (**Figure 6D**).

Collectively these results indicate that Aer administration of 2-12C significantly reduced viral load in BAL and lung but did not affect nasal shedding following direct influenza challenge.

## 4 Discussion

Identifying approaches to block transmission remains a pressing need in combatting respiratory infection, and their evaluation in a biologically relevant large animal host that is susceptible to natural infection is crucial. The possibility of rapid deployment and immediate effect in an individual makes mAbs a promising option for preventing transmission. While substantial progress has been achieved in antibody delivery, there are significant challenges in terms of high costs associated with production, purification, and quality control. Moreover, long-term protection is difficult with a single inoculation because of the inherently short half-life of mAbs. However, alternative *in vivo* Ab gene transfer strategies, including the use of DNA, RNA or viral vectors, polymer conjugation, and Fc modifications, have shown that mAbs can be stably maintained in host tissue, leading to robust and prolonged expression (7, 22, 23).

Most testing of mAbs uses small animal models to determine efficacy against direct challenge (1–5, 24). We have established a reproducible and robust pig direct influenza challenge model using the strongly neutralizing anti-HA1 mAb, 2-12C (14). Intravenous administration of 2-12C reduced nasal shedding, lung damage, and lung virus load but did not prevent virus nasal shedding following direct influenza intra-nasal challenge (7).

In order to circumvent the direct inoculation of the virus and better mimic natural infection, we developed a contact challenge model in which 2-12C treated animals were co-housed with donor pigs previously infected with pH1N1. 2-12C pre-treatment completely prevented infection and none of the treated pigs shed the virus. Two of the five untreated control pigs did not shed the virus, but four of them had the virus in the BAL, three in the lung, and histopathologically they had significantly more prominent lung pathology than the 2-12C treated group. A limitation of this direct contact influenza challenge model is that there is more variability, indicating that a larger number of animals per group should be used in future experiments as others have found in an airborne transmission model (25). Nevertheless, these results clearly show that the contact challenge model can identify potentially useful transmission-blocking treatments, and better mimics natural exposure than direct intra-nasal challenge.

To the best of our knowledge, this is the first study to test the efficacy of mAb using contact challenge in pigs. Since 2-12C prevents nasal shedding, it is most likely that the treated pigs will be unable to transmit to further naïve animals. This is in contrast to previous experiments where we tested the ability of immunized and challenged pigs to transmit to naïve animals (26). Even the most efficient vaccines, which significantly reduced viral shedding, failed to prevent transmission to naïve in-contact animals. This indicates that prevention of transmission in the pig direct contact model is very difficult. Experiments testing the transmissibility of influenza viruses by airborne or direct contact experiments also showed that airborne transmission is less efficient than contact in pigs (27). Airborne transmission studies between pigs and ferrets or pigs and ducks have also shown that airborne transmission can be blocked more easily (28, 29). Therefore, direct contact transmission experiments set a high bar for a transmission blocker, suggesting that 2-12C is a very effective treatment to prevent onward transmission, although formal proof for this would be to expose further naïve animals to the previously co-housed 2-12C recipients.

Most anti-influenza mAbs evaluated in animals or humans are given parenterally. However, the influenza virus targets epithelial cells of the upper and lower respiratory tracts. Therefore, local administration of neutralizing mAb to the target tissue may be a clinically relevant approach. However, when we compared the efficacy of 2-12C administered intravenously or by aerosol, surprisingly Aer 2-12C did not prevent shedding in the direct challenge model, although it abolished viral load in the lung and BAL. The biodistribution showed that Aer delivered nearly 3 logs less 2-12C to serum, with most being delivered to the lower respiratory tract as it was recovered in the BAL. In the lung, I.V. resulted in a higher titer than Aer, most likely because the lung is a highly vascularized organ.

In previous studies, we used the human broadly neutralizing anti-stem FI6 mAb delivered by aerosol and showed only a marginal protective effect on gross pathology (30). However, this might have been due to the inability of FI6 to bind porcine poFcγRIIIa and therefore mediate Fc functions (30–32). Aerosol delivery of nanobodies targeting human respiratory virus fusion protein reduced disease in newborn lambs (33), while inhaled nanobodies against the Spike protected hamsters against SARS-CoV-2 (34). Studies in mice have shown that the intranasal route of delivery of anti-influenza antibodies results in increased protection (35–37). Furthermore, in mice, aerosol and intranasal delivery of broadly neutralizing anti-HA stalk mAbs were prophylactically and therapeutically protective, demonstrating that a significantly less antibody can afford protection (38). In the present study, the administered aerosol dose of 2-12C was two and a half logs less compared to the I.V. dose and it may still be possible that a higher aerosol dose (still significantly less than the I.V. dose) may be effective in preventing nasal shedding.

In summary, we propose that the pig direct contact challenge model will be useful for testing transmission blockers, although confirmation that onward transmission is prevented may require co-housing with more naïve pigs. Aerosol delivery of 2-12C to the respiratory tract did not prevent nasal shedding but abolished viral load in the lung and BAL and may, therefore, be a very useful therapeutic strategy to prevent severe disease. However higher dose of aerosol or intranasal delivery may still be effective. We propose that the pig influenza model will be useful for bridging the gap between small animals and human clinical trials in testing candidate monoclonal antibodies and emerging delivery platforms.

## Data availability statement

The original contributions presented in the study are included in the article/**Supplementary Material**. Further inquiries can be directed to the corresponding author.

## Ethics statement

The animal study was approved by the Pirbright Institute and Animal and Plant Health Agency (APHA). The study was conducted in accordance with the local legislation and institutional requirements.

## Author contributions

ET and PS obtained the funding; AM, DV, PS, and ET designed the studies; AM, DV, PR, BP, RM, AT, PS, and ET performed the pig experiments and processed the samples. FL and AN performed pathological analysis; AM and BP acquired, analyzed, and interpreted the data; ET and AM wrote the manuscript. All authors contributed to the article and approved the submitted version.

## Supplementary material

Supplementary Figure 1 Lung pathology. Recombinant 2-12C was administered intravenously to pigs 24 hours prior to being placed in contact with donor pigs previously infected with pH1N1. Animals were co-housed for five days. Recipients were culled at seven days post contact; control pigs received no treatment. Lungs were scored for the appearance of gross and histopathological lesions. The scores of each individual and group means are shown **(A)**. Representative gross pathology, histopathology (H&E staining; original magnification 3100), and immunohistochemical NP staining (original magnification 3200) for each group are shown **(B)**. Pathology scores were analyzed using an unpaired t-test. Asterisks denote significant differences **p*<0.05, versus indicated control groups.

Supplementary Figure 2 Lung pathology. Recombinant 2-12C was administered intravenously or by aerosol to pigs 24 h prior to inoculation with pH1N1 virus. The animals were culled at 4 DPI, and lungs were scored for the appearance of gross, histopathological lesions and NP staining by immunohistochemistry. Representative gross pathology images of the lungs of pigs from each treatment group. The black arrowhead shows hyperemic areas of consolidation in the control animal **(A)**. Representative histopathological changes in the lung (bronchiole and alveolar spaces) using hematoxylin and eosin staining (H&E) (magnification x200), and immunohistochemical nucleoprotein (NP) staining (magnification x200) for each group are shown **(B)**. In the control group, the bronchiolar lumen appears occupied with a suppurative exudate (white arrowhead) with abundant virus labeling (brown staining cells) in bronchiolar epithelial cells and inflammatory cells. Alveolar walls are thickened (black arrowhead) and the virus (brown staining cells) can be detected in alveolar spaces.
