# Supplementary figures and images for: A direct contact pig influenza challenge model for assessing protective efficacy of monoclonal antibodies
Source: Front Immunol. 2023 Oct 27;14:1229051. doi: 10.3389/fimmu.2023.1229051 (PMC10641767; doi:10.3389/fimmu.2023.1229051)

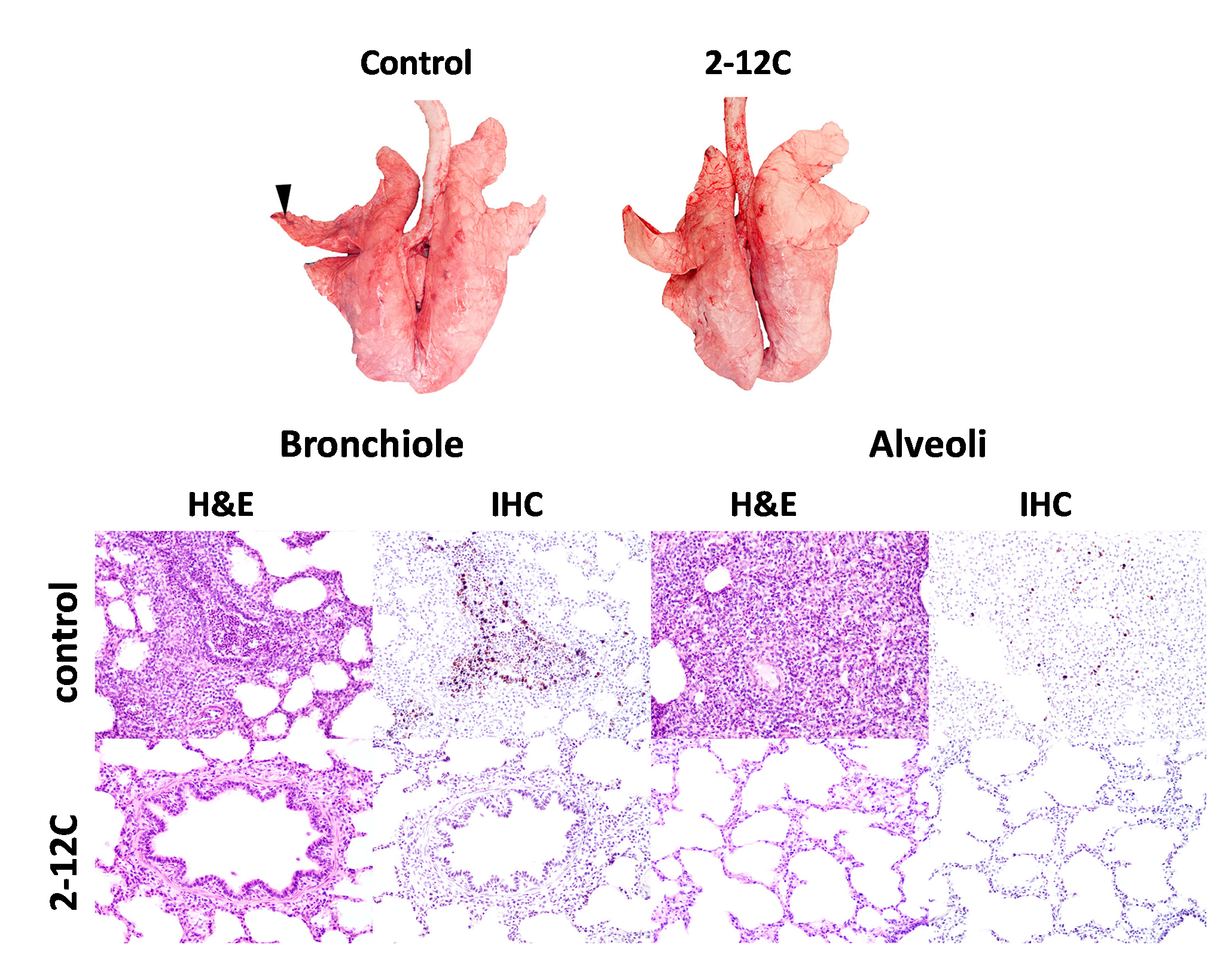

Supplement: Supplementary Figure 1 — Lung pathology. Recombinant 2-12C was administered intravenously to pigs 24 hours prior to being placed in contact with donor pigs previously infected with pH1N1. Animals were co-housed for five days. Recipients were culled at seven days post contact; control pigs received no treatment. Lungs were scored for the appearance of gross and histopathological lesions. The scores of each individual and group means are shown (A). Representative gross pathology, histopathology (H&E staining; original magnification 3100), and immunohistochemical NP staining (original magnification 3200) for each group are shown (B). Pathology scores were analyzed using an unpaired t-test. Asterisks denote significant differences *p<0.05, versus indicated control groups. [file Image_1.tif]

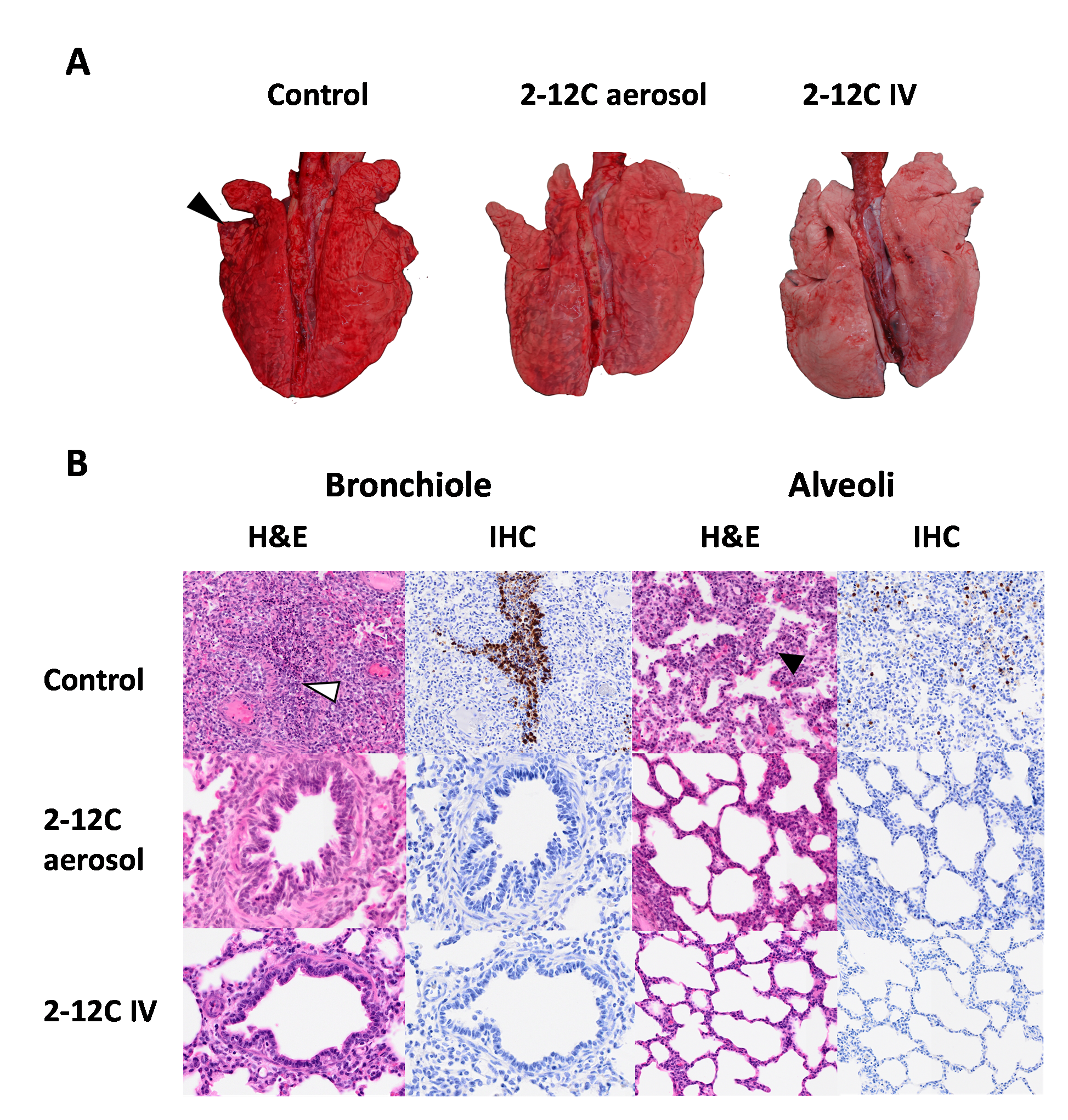

Supplement: Supplementary Figure 2 — Lung pathology. Recombinant 2-12C was administered intravenously or by aerosol to pigs 24 h prior to inoculation with pH1N1 virus. The animals were culled at 4 DPI, and lungs were scored for the appearance of gross, histopathological lesions and NP staining by immunohistochemistry. Representative gross pathology images of the lungs of pigs from each treatment group. The black arrowhead shows hyperemic areas of consolidation in the control animal (A). Representative histopathological changes in the lung (bronchiole and alveolar spaces) using hematoxylin and eosin staining (H&E) (magnification x200), and immunohistochemical nucleoprotein (NP) staining (magnification x200) for each group are shown (B). In the control group, the bronchiolar lumen appears occupied with a suppurative exudate (white arrowhead) with abundant virus labeling (brown staining cells) in bronchiolar epithelial cells and inflammatory cells. Alveolar walls are thickened (black arrowhead) and the virus (brown staining cells) can be detected in alveolar spaces. [file Image_2.tif]
